# Rod-Cone Dystrophy Related WDR34 Is Essential for Ciliary Integrity and Survival of Mammalian Photoreceptor Cells

**DOI:** 10.1167/iovs.67.1.26

**Published:** 2026-01-12

**Authors:** Rong Zou, Jinrui Cai, Lin Fan, Luning Liu, Can Chen, Guangyi Chen, Xian Yang, Kuanxiang Sun, Xianjun Zhu

**Affiliations:** 1 The Sichuan Provincial Key Laboratory for Genetic Diseases, Center for Medical Genetics, Sichuan Provincial People's Hospital, School of Medicine, University of Electronic Science and Technology of China, Chengdu, Sichuan, China; 2 Sichuan-Chongqing Joint Key Laboratory for Pathology and Laboratory Medicine, Jinfeng Laboratory, Chongqing, China; 3 Department of Ophthalmology, The Affiliated Hospital of Qingdao University, Qingdao, Shandong, China

**Keywords:** WDR34, intraflagellar transport, retinal degeneration, photoreceptor cilia, gene therapy

## Abstract

**Purpose:**

The photoreceptor outer segment is a highly specialized ciliary structure essential for phototransduction, rendering photoreceptors especially vulnerable to ciliary dysfunction. *WDR34*, a key component of the retrograde intraflagellar transport machinery, has been implicated in rod-cone dystrophy. However, the pathogenic mechanisms linking *WDR34* deficiency to photoreceptor degeneration remain elusive. In this study we aim to investigate the in vivo function of *Wdr34* in the photoreceptor cells using conditional knockout allele of *Wdr34*.

**Methods:**

We generated retina-specific *Wdr34* knockout mice using rod-specific and cone-specific drivers to investigate the in vivo roles of WDR34 in photoreceptor maintenance.

**Results:**

*Wdr34* deficiency in rod photoreceptors resulted in progressive rod cell degeneration accompanied by a marked decline in scotopic electroretinography (ERG) responses. Similarly, cone-specific *Wdr34* ablation led to impaired photopic ERG responses and subsequent cone photoreceptor death. Transcriptomic profiling of *Wdr34*-deficient retinas revealed broad differential gene expression changes, with significant enrichment in axonemal integrity and microtubule-based transport. Notably, subretinal delivery of an adeno-associated virus vector expressing WDR34 significantly preserved rod photoreceptor structure and function, underscoring the therapeutic potential of *WDR34* gene supplementation.

**Conclusions:**

Our findings establish WDR34 as a critical factor for photoreceptor survival and function, emphasizing its role in the pathogenesis of WDR34-associated retinal degeneration. Moreover, this study demonstrates that WDR34-targeted gene therapy can effectively delay photoreceptor loss, highlighting a promising treatment strategy for patients with WDR34-related retinal disease.

Retinal photoreceptors are highly specialized neurons composed of an outer segment (OS), inner segment (IS), cell body, connecting cilium (CC), and synaptic terminal. OS is a highly modified sensory cilium that detects light through its stacked membranous discs densely packed with phototransduction proteins. The CC exclusively serves as the structural bridge linking the IS to the base of the OS, while the ciliary axoneme extending from the CC penetrates approximately two thirds into the OS.^1^^,^^2^ Like all cilia, the OS lacks intrinsic biosynthetic machinery, because all protein-synthesizing organelles reside in the IS. Consequently, the tightly regulated trafficking of proteins from the IS to the OS rely exclusively on the CC.^2^ Furthermore, the entire OS structure is completely renewed approximately every 10 days. This process requires significant protein synthesis to maintain its structure and function, highlighting the critical role of CC in protein transport. Primary ciliary dysfunction is increasingly linked to a growing number of nonsyndromic retinal dystrophies, such as retinitis pigmentosa (RP).^3^ RP is a severe retinal degeneration disease,^4^ characterized primarily by the primary degeneration of photoreceptors.^5^ In the mid to late stages, they experience night blindness and progressive visual field loss, eventually developing tunnel vision and ultimately leading to blindness,^6^ affecting over 1.5 million patients worldwide.^7^

A recent study reported the identification of a WDR34 mutation through genomic sequencing in a patient with non-syndromic rod-cone dystrophy, which was proposed as the likely causative variant. However, the precise pathogenic mechanism remains unclear.^8^ WDR34, also known as dynein-2 intermediate chain 2, is a component of the cytoplasmic dynein-2 complex involved in retrograde intraflagellar transport (IFT) within primary cilia.^9^ At the tip of cilia, the IFT comprises three multi-protein subcomplexes, namely IFT-A, IFT-B, and BBSome, which carry cytoplasmic and membrane-bound cargo proteins. The IFT-dynein can convert anterograde complexes into retrograde complexes, pushing back the BBS. Therefore WDR34 plays a significant role in ciliogenesis and IFT protein transport,^10^ and its interaction with light chains is crucial for the dynein 2–mediated retrograde transport.^11^ Previous studies on *WDR34* reported that mutations in WDR34 led to Jeune syndrome or asphyxiating thoracic dystrophy,^12^ short-rib polydactyly syndrome type III/severe asphyxiating thoracic dysplasia, and can also inhibit the transforming growth factor-β–activated kinase 1 in the NF-κB activation pathway.^13^ It can activate the Wnt/Beta-Catenin signaling pathway in liver cancer cells^14^ and impact the Hedgehog signaling pathway in ciliogenesis.^15^ Collectively, these studies provide compelling evidence that WDR34 plays a critical role in human disease pathogenesis.

In this study, we aimed to elucidate the role of *Wdr34* in retinal function and degeneration by generating rod- and cone-specific *Wdr34* knockout mice. We sought to determine whether *Wdr34* is essential for photoreceptor survival and function and to explore the underlying molecular mechanisms. Furthermore, we evaluated the potential of gene supplementation as a therapeutic strategy for WDR34-associated retinal degeneration. Our findings reveal novel insights into the contribution of *Wdr34* to ciliary integrity and phototransduction, supporting its importance in maintaining retinal health and suggesting new avenues for treatment.

## Methods

### Generation of *Wdr34* Knockout Models and Genotyping

All animal experiments were approved by the Institutional Animal Care and Use Committee of the Sichuan Provincial People's Hospital (Chengdu, Sichuan, China) and were conducted in accordance with the ARVO Statement for the Use of Animals in Ophthalmic and Vision Research. The experimental mice were housed in the SPF-grade animal facility of Sichuan Provincial People's Hospital. This facility is equipped with individually ventilated cages and maintained under stringent hygienic conditions.

*Wdr34^flox/+^* transgenic mice in a C57BL/6J background were generated by Cyagen Biosciences (Suzhou, China). Mice with a *Wdr34* deletion, specifically in retinal rod or cone cells, were generated using the Cre-loxP system. CRISPR/Cas9 technology was utilized to insert loxP sequences into the two sides of Exon3 of the *Wdr34* gene to generate *Wdr34^flox/flox^* mice. Then, *Wdr34^flox/flox^* mice were crossed with Rod-Cre mice (purchased from The Jackson Laboratory, USA) or HRGP-Cre mice (a gift from Prof. Yun-zheng Le, University of Oklahoma Health Sciences Center, Oklahoma City, OK, USA) to obtain F1 progeny (*Wdr34^flox/+^*; Cre). Subsequently, F1 mice were crossed with *Wdr34^flox/flox^* mice to produce *Wdr34^flox/flox^*; Rod-Cre mice (*Wdr34^RKO^* mice) or *Wdr34^flox/flox^*; HRGP-Cre mice (*Wdr34^HKO^* mice). Negative control mice were *Wdr34^flox/flox^*.

Mouse genomic DNA samples were extracted from mouse toes and genotyped using PCR. The floxed *Wdr34* alleles and Rho-Cre or HRGP-Cre were genotyped using the corresponding primers. The PCR products were separated by DNA electrophoresis on a 3% agarose gel. The primers used are listed in Supplementary Table S1.

### RNA Isolation, RT-PCR and RT-qPCR

Total RNA was extracted from mouse retinal tissues or cells using the TransZol Up kit (Cat. no. T11L01; TransGen Biotech, Beijing, China). CDNA was synthesized by reverse transcription using TransScript All-in-One First-Strand cDNA Synthesis SuperMix for qPCR (cat. no. S10111, TransGen Biotech). Quantitative PCR was performed using PerfectStart Green qPCR SuperMix (cat. no. R20719; TransGen Biotech) and a 7500 Fast Real-Time PCR Detection System (cat. no. B4351104; ThermoFisher, St. Louis, MO, USA). Target gene expression levels were normalised to GAPDH mRNA levels. Primers were designed using Primer3Plus: Supplementary Table S2 shows the specific primer sequences.

### Electroretinography

For electroretinographic evaluation of *Wdr34^RKO^* mice and *Wdr34^HKO^* mice, mice were anesthetized with chlorpromazine (80 mg/kg) and ketamine (16 mg/kg) administered intraperitoneally in saline solution at night following dark adaptation for at least eight hours. The apparatus used was the Celeris, a visual electrophysiological assessment device manufactured by Wuhan Shibo Medical Equipment Co., Ltd (Wuhan, China). A drop of tropicamide was administered to the eyes of the mice, and the dark adaptation signals were recorded sequentially at light intensities of 0.02, 0.2, 2 and 20 cd-s/m^2^. Signs were recorded at light intensities of 3 and 10 cd-s/m^2^ for light adaptation measurements over five minutes. The signals were sampled at 0.8 ms intervals and subsequently averaged.

### Histological Analysis

For hematoxylin and eosin staining (H&E), mice of varying months of age (three knockout mice and three control mice, all males) were obtained, euthanized, and the eyeballs were extracted and positioned in the nasal markers in FAS solution (0.08 M phosphate buffer containing 1.22% glutaraldehyde and 0.8% paraformaldehyde) overnight. On the following day, the eyeballs were paraffin-embedded (in the same direction according to the marker embedding) and then cut to a thickness of approximately 5 µm. According to standard protocols, sections containing the optic nerve (ON) were selected for H&E staining. The final outer nuclear layer (ONL) thickness was measured three times and averaged.

### Immunohistochemistry

For retina sections, following neck dissection, the eyeballs were immersed in a 4% paraformaldehyde solution and fixed at 4° for 2 hours. Subsequently, a 30% sucrose solution was added, and the eyeballs were dehydrated at 4°C for 2 hours. The lens was excised, and the eyeballs were embedded in optimal cutting temperature compound, frozen at −20°C, and then sectioned at a thickness of 12 µm. The sections were incubated in an oven at 37°C for 45 minutes, blocked and permeabilized with PBS solution containing 5% FBS and 0.1% Triton X-100, and incubated with the primary antibody overnight at 4°C. The slices were stained with Alexa Fluor 594/488 conjugated goat anti-mouse/rabbit secondary antibody (1:500 dilution; cat. no. A1108 and A11005; Invitrogen, Carlsbad, CA, USA) and DAPI for two hours at room temperature. After sealing the slices with a sealing oil resistant to fluorescence quenching, images were taken on a Zeiss LSM 900 confocal scanning microscope (Zeiss, Oberkochen, Germany).

### Retina Flat-Mount

As for flat-mount retina, following the execution of the mice, the eyes were immersed in a 4% paraformaldehyde solution and fixed at 4°C for two to four hours. The cornea was clipped, the lens was removed, and the sclera and retinal pigment epithelium were stripped. After cutting the retina to a four-leaf clover shape, the retina was fixed with ice-cold methanol. The retina was closed and permeabilized with a PBS solution containing 10% FBS and 0.2% Triton X-100. The primary antibody was incubated at 4°C overnight. The retinas were then stained with Alexa Fluor 488-conjugated goat anti-rabbit secondary antibody and Alexa Fluor 594-conjugated peanut agglutinin (1:500 dilution; cat. no. RL1072; Vector Laboratories, Burlingame, CA, USA) for two hours at room temperature. After the sealing of the sections with anti-fluorescence quenching sealing oil, images were captured on a Zeiss LSM 900 confocal scanning microscope. Supplementary Table S3 lists the antibodies used in the experiments.

### Immunocytochemistry

The cell lines were seeded in a 24-well plate containing cover glass and cultivated until the cell density reached approximately 90%. The medium should then be aspirated, and the plate fixed in a 4% paraformaldehyde solution for 20 minutes at room temperature. The plate should then be washed three times for five minutes with a PBS buffer. The plate should then be sealed and permeabilized with a PBS solution containing 5% FBS and 0.1% Triton X-100 for one to two hours at room temperature. The primary antibody should then be added to the plate and left overnight at 4°C. The cells were incubated with Alexa Fluor 594/488-conjugated goat anti-mouse/rabbit secondary antibody for two hours at room temperature. The cell crawls were then sealed with anti-fluorescence quenching sealing oil, and images were captured on a Zeiss LSM 900 confocal scanning microscope. Supplementary Table S3 lists the antibodies used in the experiments

### Western Blotting

The cells or tissues were lysed in a standard RIPA lysis buffer (Cat# R0010, Solabio, China), which contained a protease inhibitor (cat. no. 11,697,498,001; Roche, Redwood City, CA, USA) and a phosphatase inhibitor (cat. no. 4,906,845,001; Roche). The protein concentrations were determined using the Enhanced BCA Protein Assay Kit (cat. no. P0010S; Beyotime Biotechnology, Jiangsu, China), and equal amounts of protein were separated on SDS-polyacrylamide gels and transferred to NC membranes. The membranes were incubated for two hours at room temperature in a solution of Tris-HCl containing 8% skim milk and Tween 20 (TBST buffer). The membranes and primary antibodies were incubated overnight at 4°C (the primary antibody was prepared in TBST buffer containing 8% skim milk). Signal acquisition was performed using SuperSignal West Pico Chemiluminescent Substrate (cat. no. 34,577; Thermo Fisher Scientific, Waltham, MA, USA) after two hours of incubation at room temperature using HRP-conjugated mouse/rabbit secondary antibodies (1:5000; Bio-Rad, Hercules, CA, USA). Signal intensity was analyzed using ImageJ software and normalized with GAPDH. Supplementary Table S3 lists the antibodies used in the experiments.

### Mouse Subretinal Injection

The adeno-associated virus serotype 8 used in this experiment was constructed by Shanghai Genechem Co.Ltd (Shanghai, China). The three-week-old mice were anesthetized with an intraperitoneal injection of chlorpromazine (80 mg/kg) and ketamine (16 mg/kg) in normal saline solution, and the pupils were dilated with 0.5% compound tropicamide eye drops. The mice were placed on a microsurgical manipulator, and a 30-gauge needle was used to create a vertical incision 1 mm posterior to the scleral margin of the angle. A 5-µL microsyringe (to aspirate 1.5 µL of adeno-associated virus overexpressing WDR34) was injected through the incision until the needle was pressed against the contralateral retina. Subsequently, 0.3% levofloxacin ophthalmic ointment was administered topically to the eye to prevent corneal desiccation and infection. All instruments that had been in contact with the virus were rendered inactivated to ensure the safety of the experiment.

### RNA-Seq Analysis

Retinal tissues were obtained from two-month-old mice (four wild-type and four knockout mice, all male) and sent to Shanghai Biozeron Biotechnology Co., Ltd. (Shanghai, China) for transcriptomic testing. After initial total RNA extraction from the samples, ribosomal RNA was removed using a standard kit to enrich messenger RNA (mRNA). The enriched mRNA was then reverse-transcribed into double-stranded cDNA. After end repair, adapters were ligated to the cDNA, and the library was finally prepared via PCR amplification for sequencing.

The sequencing library was constructed using the Illumina TruseqTM RNA sample prep Kit. Briefly, eukaryotic mRNA with polyA tails was enriched using oligo(dT)-coupled magnetic beads. The purified mRNA was then fragmented via ultrasonication. Using these fragmented mRNAs as templates and random oligonucleotides as primers, the first strand of cDNA was synthesized in an M-MuLV Reverse Transcriptase system. The RNA strand was subsequently degraded by RNase H, and the second cDNA strand was synthesized using DNA polymerase I with dNTPs. The purified double-stranded cDNA underwent end repair, was adenylated at the 3' ends, and was ligated with sequencing adapters. cDNA fragments of approximately 200 bp were selected using AMPure XP beads, followed by PCR amplification. The PCR products were purified again with AMPure XP beads to yield the final library. After passing quality control, libraries were pooled based on their effective concentrations and the desired sequencing data volume for next-generation sequencing.

After sequence comparison and quantification, bowtie was used to compare the sequencing data, following quality control with the reference genome, and quantify the expression of each gene. Finally differentially expressed gene analysis and pathway enrichment analysis: Utilize DESeq2 to identify differentially expressed genes (|log_2_foldchange| ≥ 0.5) for gene ontology analysis and KEGG pathway enrichment analysis. Ultimately, a *P* value less than or equal to 0.05 was deemed significant.

### Statistical Analysis and Image Processing

Immunofluorescence staining experiments were conducted using a Zeiss LSM900 and analyzed with the Zeiss Zen blue edition software. Protein immunoblotting experiments were analyzed with ImageJ software, while fluorescence quantitative PCR experiments were analyzed with ABI7500 software. All data were subjected to statistical analysis and processing using GraphPad Prism 9.0 software. The statistical differences were calculated using either the independent samples *t*-test or Tukey's multiple comparisons ANOVA (*P* < 0.05).

## Results

### Cone-Specific Ablation of *Wdr34* Results in Cone Photoreceptor Defects and Visual Dysfunction

To investigate the role of the *Wdr34* gene in cone photoreceptors, we employed transgenic mice carrying the human *OPN1LW* promoter (HRGP)-driven Cre recombinase system (HRGP-Cre).^16^ These mice were then crossed with *Wdr34*^flox/flox^ mice (containing LoxP sites flanking exon 3) to successfully establish a cone-specific *Wdr34* knockout mouse model (hereafter referred to as HKO; Supplementary Fig. S2A). Genotypic validation confirmed successful recombination via detection of a Cre-specific amplicon (Supplementary Fig. S2B).

To assess visual function, we performed photopic electroretinography (ERG) on four-month-old mice (Supplementary Fig. S2C). HKO mice exhibited significantly reduced a-waves and b-waves at flash intensities of 3.0 cd · s/m^2^ and 10.0 cd · s/m^2^ (Supplementary Figs. S2D, S2E). Immunohistochemical analysis of retinas from eight-month-old HKO mice using antibodies against L/M-opsin and cone arrestin, along with peanut agglutinin labeling, revealed pronounced cone loss (Supplementary Figs. S2F, S2G). Consistent with these observations, retinal whole-mount staining confirmed a substantial reduction in L/M-opsin-positive photoreceptors (Supplementary Fig. S2H).

### Rod-Specific Deletion of *Wdr34* Leads to Photoreceptor Degeneration and Visual Function Decline

**Figure 1.**
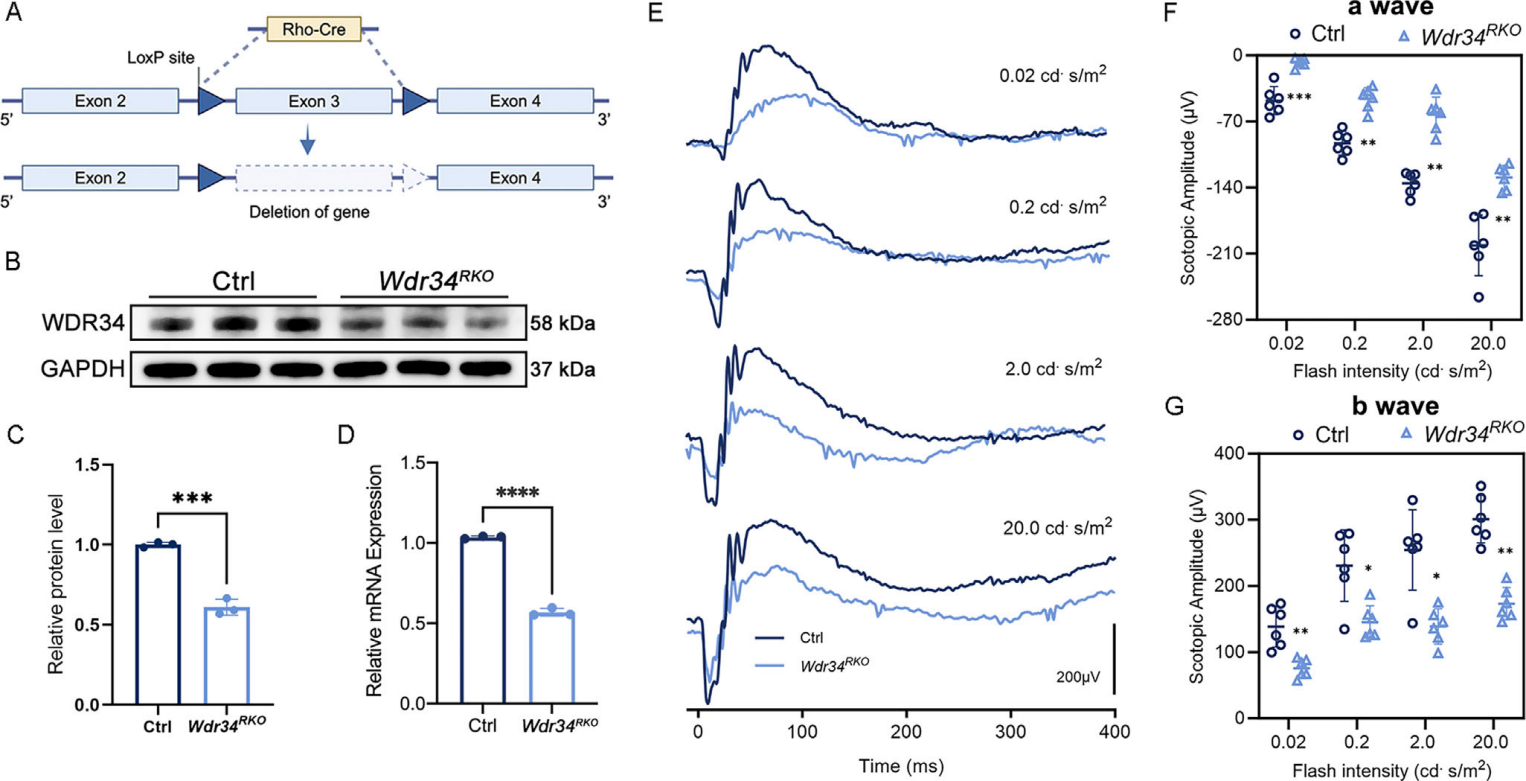
Specific deletion of *Wdr34* in retinal rods resulted in impaired visual function. **(A)** Schematic diagram illustrating the generation of *Wdr34^RKO^* conditional knockout mice. **(B, C)** Western blot **(B)** and quantitative comparison **(C)** of WDR34 protein expression in retinas from *Wdr34^flox/flox^* (Ctrl) and *Wdr34*^flox/flox^; *Rho-Cre* (*Wdr34^RKO^*) mice (*n* = 3). GAPDH served as the loading control. **(D)** Quantitative analysis of *Wdr34* gene mRNA expression levels in the retina of *Wdr34^RKO^* mice (*n* = 3) indicates a 48% reduction in WDR34 knockout efficiency. **(E)** Representative scotopic ERG traces corresponding to flash intensities of 0.02, 0.2, 2, and 20 cd · sec/m² in two-month-old mice. **(F, G)** Statistical analysis was performed for the amplitudes of the a-wave **(F)** and b-wave **(G)** under scotopic conditions (*n* = 6, two-way ANOVA followed by Tukey's post hoc test). *P < 0.05; **P < 0.01; ***P < 0.001; ns = no significant difference. Data are presented as mean ± SD.

To elucidate the role of *Wdr34* in rod photoreceptors, we generated rod-specific *Wdr34* knockout mice (hereafter termed RKO) by crossing *Wdr34^flox/flox^* mice with transgenic mice expressing Cre recombinase under the control of the rod-specific rhodopsin (*Rho*) promoter (Rho-Cre;^17^) (Fig. 1A). Western blot (Figs. 1B, 1C, Supplementary Figs. S1A, S1B) and RT-qPCR (Fig. 1D) analyses confirmed a reduction in WDR34 expression by approximately 40% and 48%, respectively. Considering that rods constitute approximately 60% of all retinal cells in mammals.^18^

**Figure 2.**
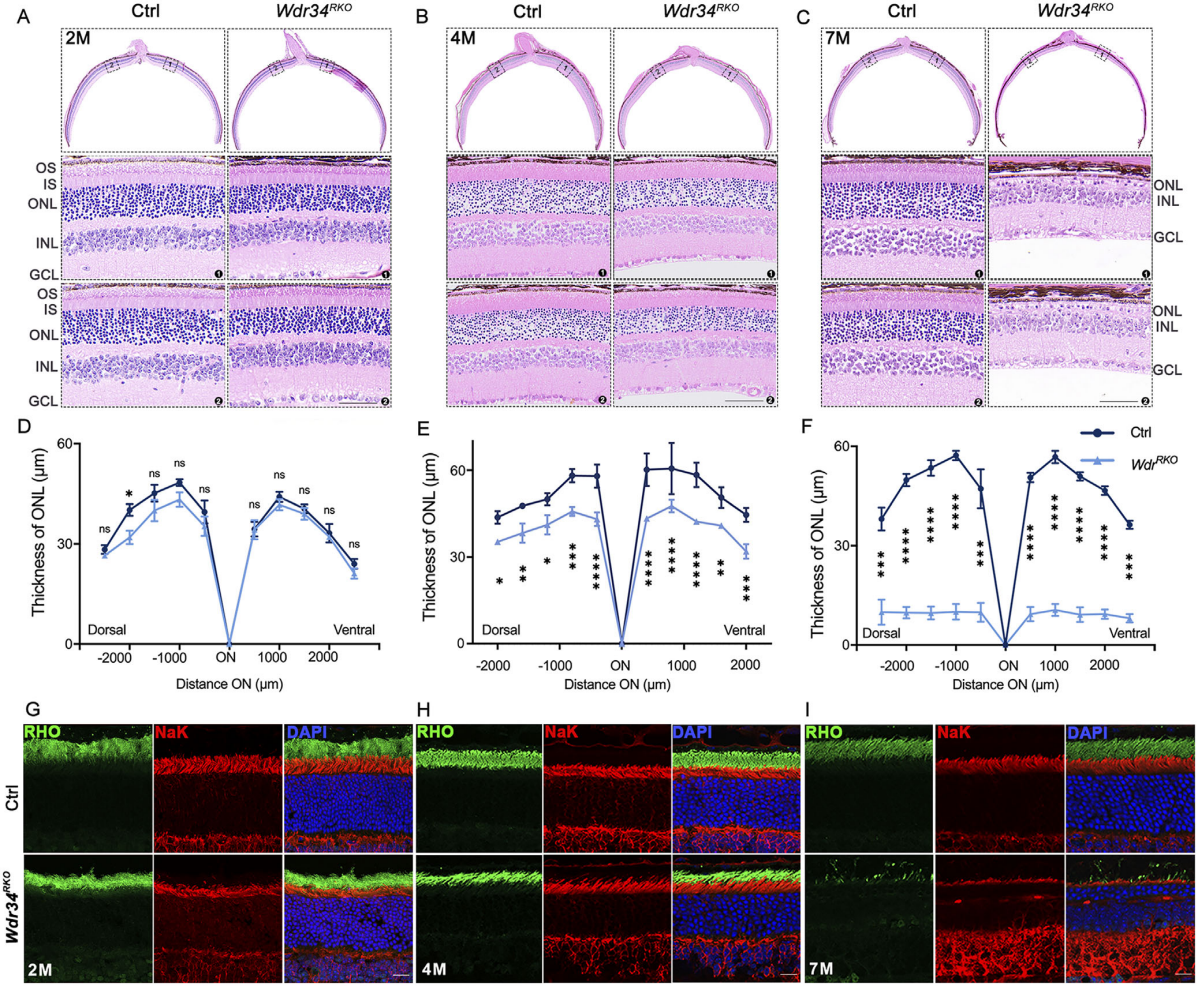
Loss of *Wdr34* led to progressive changes in mouse retina. **(A–C)** H&E staining of paraffin sections of RKO and corresponding control retinas at the ages of 2 **(A)**, 4 **(B)**, and seven months **(C)**. *Scale bar*: 25 µm. The *lower panel* depicts the quantitative analysis of the ONL thickness of the Ctrl (*n* = 5) and RKO (*n* = 5) retinas of mice at various ages. Statistical analysis was performed using two-way ANOVA, followed by Tukey's post-hoc test. **(D–F)** Retinal cryosections from two-month-old mice **(D)**, four-month-old mice **(E)**, and seven-month-old mice **(F)** were labeled with rhodopsin (*green*) and the IS marker Na–K ATPase (*red*). DAPI was used to counterstain the nuclei. *Scale bars*: 25 µm. INL, inner nuclear layer; GCL, ganglion cell layer. *P < 0.05; **P < 0.01; ***P < 0.001; ****P < 0.0001. Data are presented as the mean ± SD.

We evaluated the physiological impact of WDR34 deficiency on rod function. Scotopic ERG recordings in two-month-old RKO mice showed markedly diminished a-wave and b-wave amplitudes across a range of flash intensities compared to controls (Figs. 1E–G). Histological examination via H&E staining revealed progressive thinning of the ONL, with significant degeneration evident at four months and near-complete loss by seven months of age (Figs. 2A–C).

**Figure 3.**
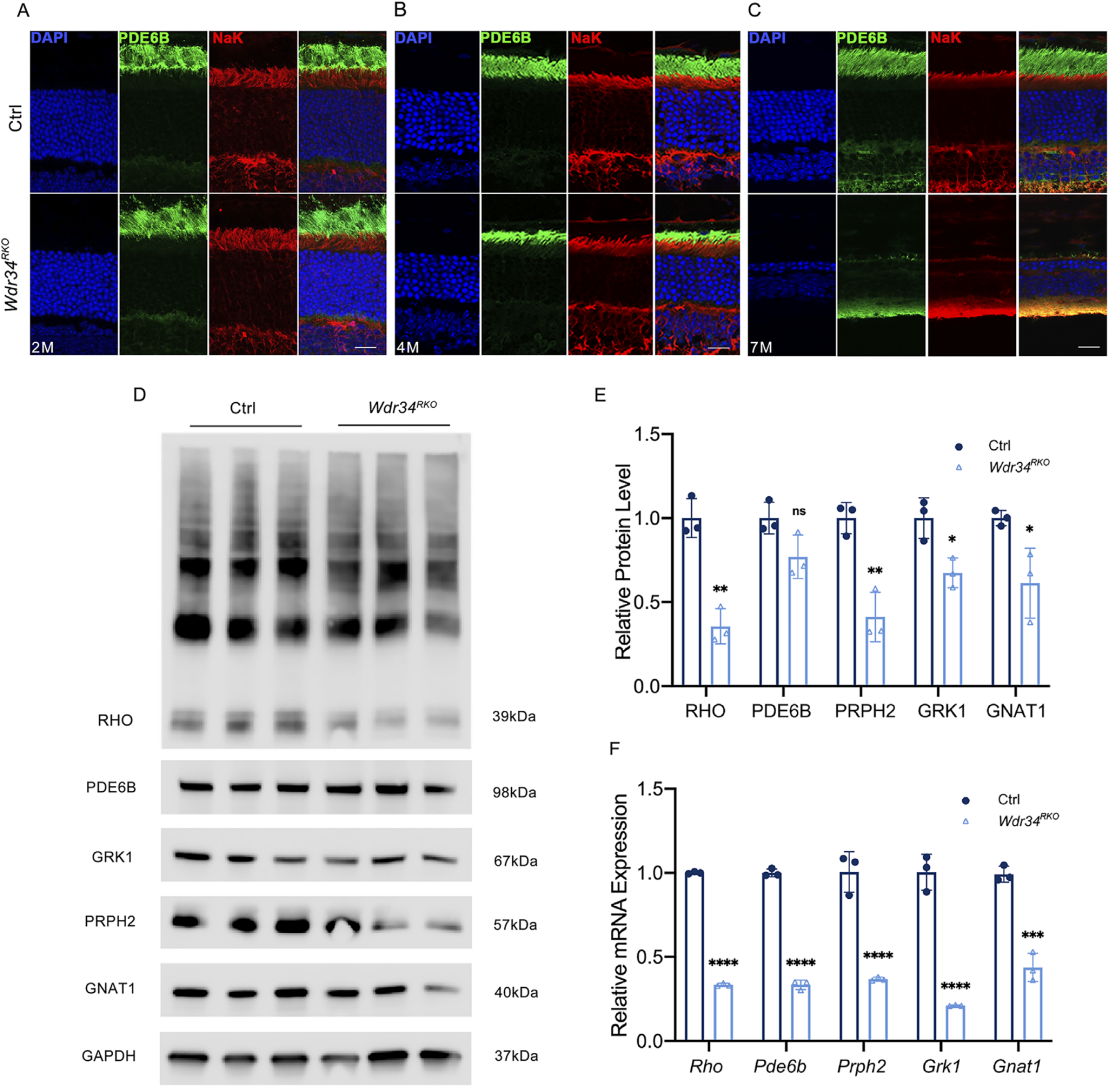
Deletion of *Wdr34* led to reduced expression levels of phototransduction-related proteins. **(A–C)** Immunofluorescence labeling of retinal cryosections from Ctrl and RKO mice at the age of two **(A)**, four **(B)**, seven **(C)** months using Pde6b (*green*) and Na–K ATPase (*red*). DAPI (*blue*) was used to counterstain the nuclei. *Scale bar*: 25 µm. **(D, E)** Western Blot **(D)** and quantitative comparison **(E)** of protein expressions of Pde6b, Grk1, Prph2, Gnat1 and Rho in retinas from two-month-old RKO mice. GAPDH served as the loading control (*n* = 3). **(F)** Quantitative analysis of *Pde6b, Grk1, Prph2, Gnat1* and *Rho* gene mRNA expression levels in the retina of *Wdr34^RKO^* mice (*n* = 3) indicated varying degrees of reduction compared to Ctrl. All comparisons were performed using Dunnett's multiple comparison test. *P < 0.05; **P < 0.01; ***P < 0.001; ****P < 0.0001. Data are presented as the mean ± *SD*.

Phototransduction relies on disc-associated proteins that are crucial for retinal function. Abnormal expression or dysfunction of these proteins can lead to the degeneration of photoreceptor cells. Immunostaining for the disc-specific protein rhodopsin (RHO) indicated shortened OS in two-month-old RKO mice, with further shortening at 4 months and near absence by seven months (Figs. 2D–F). Immunostaining for phosphodiesterase-6-β corroborated these findings (Figs. 3A–C).

To further investigate the cause of OS degeneration, we performed Western blot analysis on retinal tissues from two-month-old RKO mice. The results of Western blot and RT-qPCR demonstrated significant downregulation of key OS proteins and their transcripts in RKO retinas (Figs. 3D–F). These results indicate that *Wdr34* deficiency disrupts OS protein expression and compromises rod survival and function.

### *Wdr34* Deficiency Impairs Cilium Structure In Vivo and Vitro

**Figure 4.**
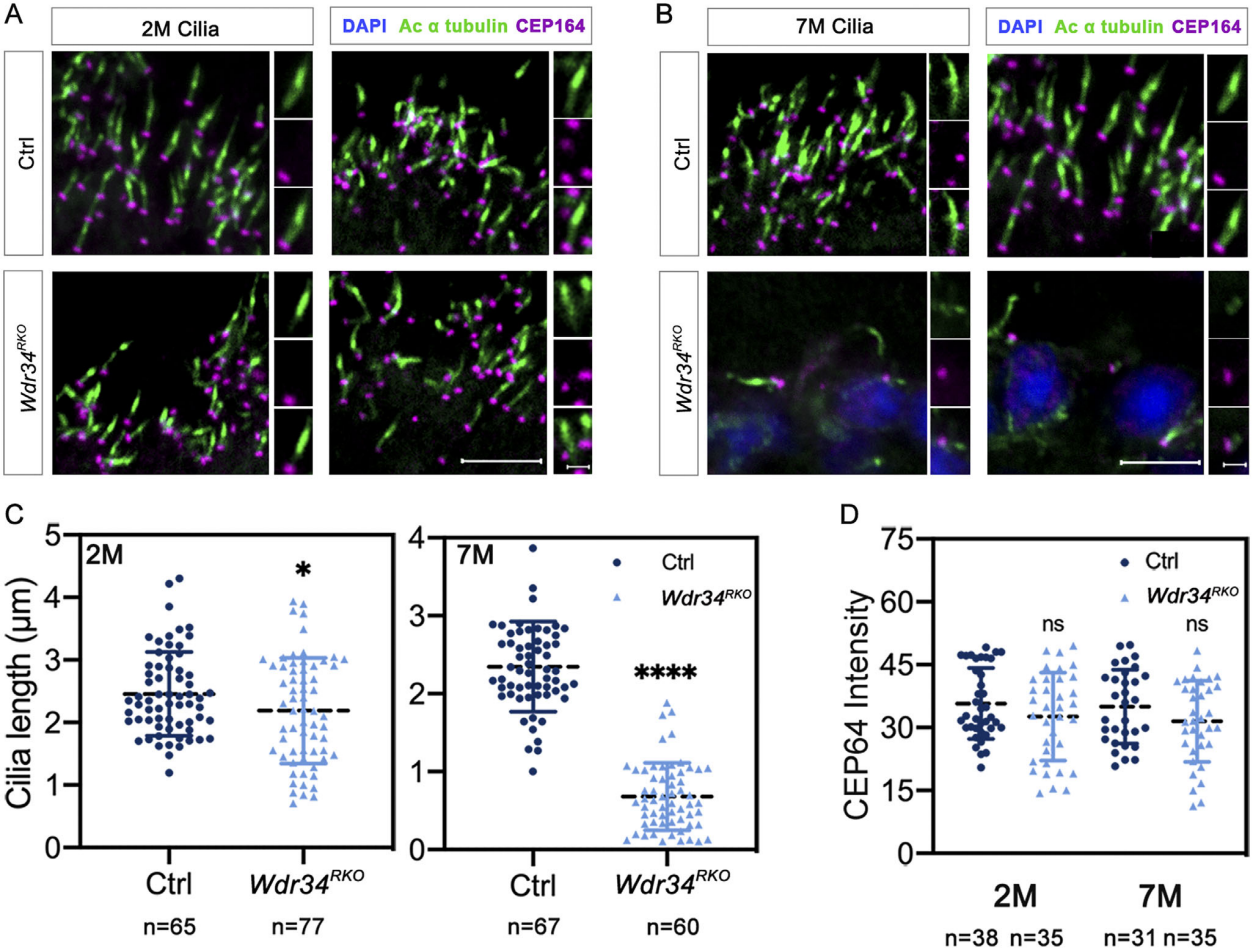
*Wdr34* deletion resulted in a decrease in the quantity and length of photoreceptor cilia. **(A)** Retinal cryosections from two-month-old mice were labeled with the cilia marker acetylated α-tubulin (Ac-tubulin, *green*) and CEP164 (*purple*). DAPI was used to counterstain the nuclei. *Scale bars*: 5 µm. The *right panels* show high-magnification images of a cropped and zoomed area. *Scale bars*: 1 µm. **(B)** Retinal cryosections from seven-month-old mice were labeled with the cilia axoneme marker acetylated α-tubulin (Ac-tubulin, *green*) and CEP164 (*purple*). DAPI was used to counterstain the nuclei. *Scale bars*: 5 µm. The right panels show high-magnification images of a cropped and zoomed area. *Scale bars*: 1 µm. **(C)** Quantification of CC (Ac-tubulin staining) lengths at different ages. **(D)** Quantification of fluorescence intensities of CEP164 in retinal cryosections. Two-way ANOVA was used for statistical analysis, followed by Tukey's post hoc test. *P < 0.05; ***P < 0.001. Data are presented as the mean ± *SD*.

The CC serves dual biological functions—providing structural support for the OS while serving as a molecular conduit for the transport of visual proteins from the IS to the OS. Given the role of WDR34 as an essential component of the dynein-2 complex in retrograde IFT, we hypothesized that its loss would affect CC integrity. Therefore we analyzed the number and length of the CC by immunohistochemical staining using an antibody against acetylated α-tubulin (Ac-tubulin). The results showed that two-month-old RKO mice exhibited a reduction in the length of photoreceptor cilia, whereas seven-month-old RKO mice displayed a further decrease in ciliary length. (Figs. 4A–C). Immunostaining with anti-CEP164 antibody demonstrated no significant alterations in basal body morphology or integrity (Figs. 4A, 4B, 4D). These results collectively indicate that *Wdr34* deficiency specifically impairs the structural integrity of photoreceptor connecting cilia but likely without affect basal body organization.

**Figure 5.**
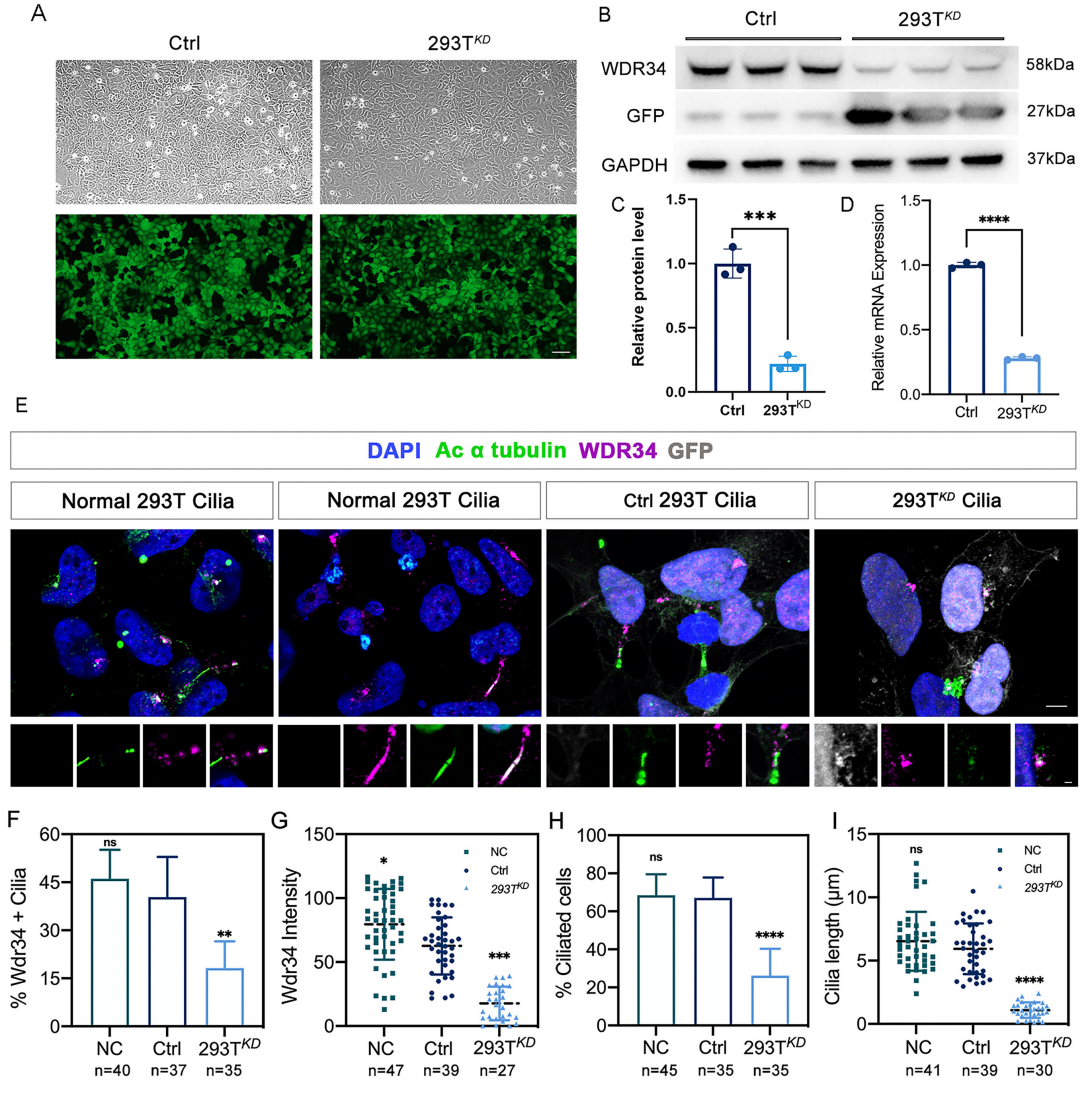
*Wdr34* knock-down HEK-293Ts exhibited drastic reduction in cilia length and in the number of cilia. **(A)** Lentivirus infection of HEK-293T. Under differential interference contrast microscopy (*white*), cells are observed to be densely covered and lentivirus GFP fluorescence (*green*) labeling reaches 90%. *Scale bar*: 500 µm. **(B, C)** Immunoblotting **(B)** and quantitative analysis **(C)** show successful knockdown of WDR34 in HEK-293T (*n* = 3). GAPDH was used as the loading control. **(D)** Quantitative analysis of relative mRNA of WDR34 gene (*n* = 3). GAPDH was used as an internal reference. **(E)** Representative immunocytochemistry images of Ac-α-tubulin (*green*) and WDR34 (*purple*). DAPI (*blue*) was used to counterstain the nuclei. *Scale bar*: 5 µm, 1 µm. **(F)** Statistical analysis of WDR34 labeling proportion in HEK-293T cells. **(G)** Quantification of fluorescence intensity of WDR34. **(H)** Statistical analysis of ciliated proportion in HEK-293T cells. **(I)** Quantification of CC (Ac-tubulin staining) lengths. Two-way ANOVA was used for statistical analysis, followed by Tukey's post hoc test. *P < 0.05; **P < 0.01; ***P < 0.001; ****P < 0.0001. Data are presented as the mean ± *SD*.

To further examine the role of *Wdr34* in ciliary maintenance, we knocked down *Wdr34* in HEK293T cells via lentiviral delivery of shRNA, achieving 90% transduction efficiency (Fig. 5A) and ∼80% knockdown at both protein and mRNA levels (Figs. 5B–D). *Wdr34* knockdown significantly reduced both the percentage of ciliated cells and cilium length (Figs. 5E–I). These results further demonstrate the crucial role of *Wdr34* in maintaining ciliary structure.

### *Wdr34* Deletion Disrupts Motor Protein Levels

To comprehensively investigate the molecular mechanisms underlying *Wdr34* deficiency-induced retinal degeneration, we performed transcriptome sequencing analysis on retinal tissues from 1.5-month-old control mice and RKO mice at the initial stage of retinal degeneration.

**Figure 6.**
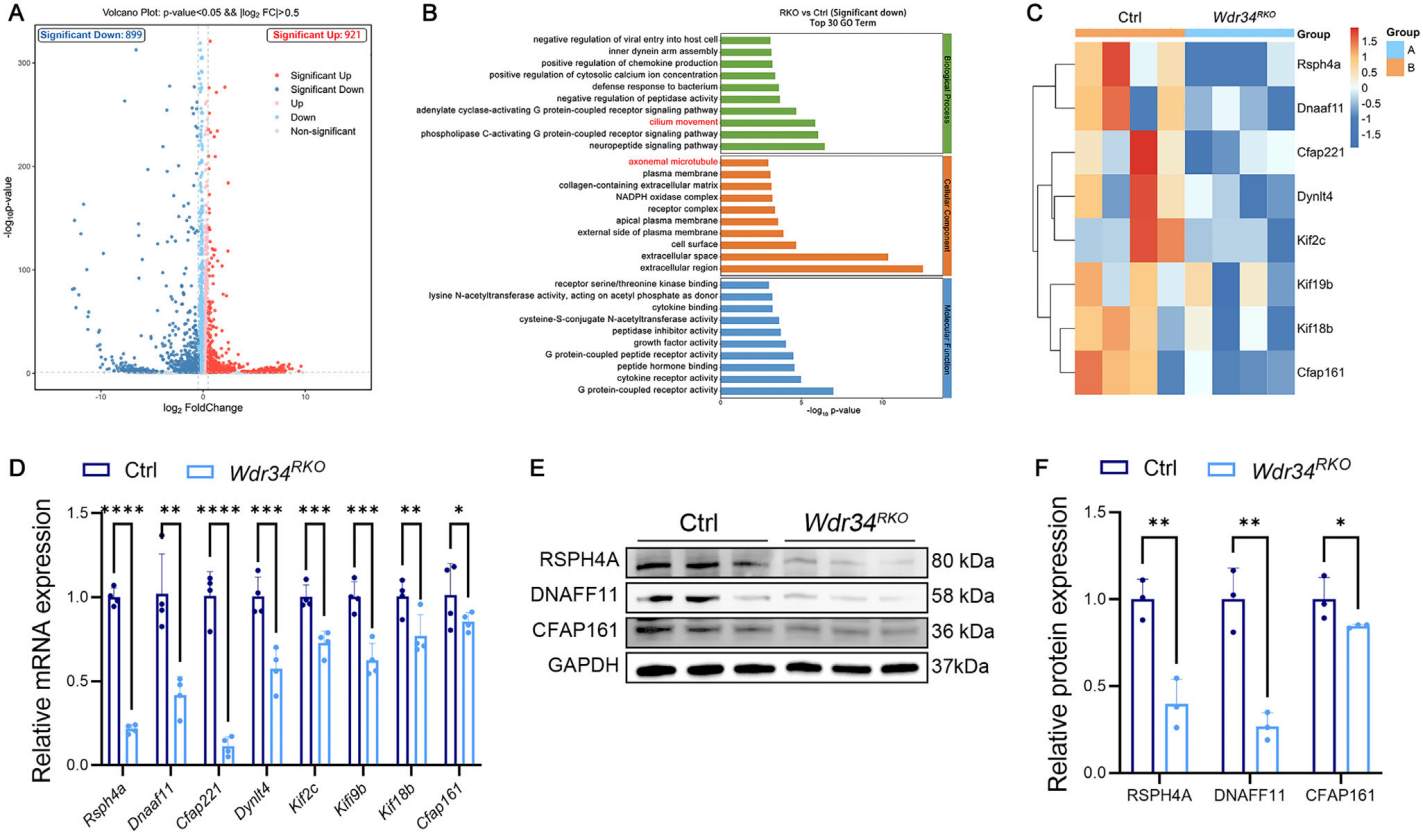
Impact of *Wdr34* knockout on motor protein and retinal metabolic levels. **(A)** Volcano plot of differentially expressed genes in the transcriptome following *Wdr34* gene knockout. *Red dots* represent upregulated genes and *blue dots* represent downregulated genes. The analysis threshold is *P* < 0.05, with a |Log_2_FoldChange| > 0.5. **(B)** Gene ontology functional enrichment analysis of downregulated genes in the transcriptome. The analysis threshold is *P* < 0.05, with a |Log_2_FoldChange| > 0.5. **(C)** Heat map of eight genes closely related to ciliary structure. **(D)** qPCR verification of mRNA expression levels of eight candidate genes in two-month-old mice retina. **(E, F)** Western blot **(E)** and quantitative comparison **(F)** of RSPH4A, DNAAF1, and CFAP161 protein expression in two-month-old mice retina (*n* = 3). GAPDH served as the loading control. *P < 0.05; **P < 0.01; ***P < 0.001; ****P < 0.0001. Data are presented as the mean ± SD.

Comparative analysis identified 899 down-regulated and 921 up-regulated genes in RKO mice (|log_2_foldchange| ≥ 0.5; *P* < 0.05) (Fig. 6A). Gene ontology enrichment analysis of these 899 down-regulated genes revealed that a subset of them were associated with cilium movement, axonemal microtubule (Fig. 6B, highlighted in red). We screened eight genes (*Rsph4a*, *Dnaaf11*, *Cfap221*, *Dynlt4*, *Kif2c*, *Kif19b*, *Kif18b*, *Cfap161*) closely related to cilia structural integrity and microtubule transport among the significantly downregulated genes, and the heat map shows these eight genes and their expression levels (Fig. 6C). We further verified the mRNA expression levels of these eight candidate genes by qPCR and found that the expression of these genes were all significantly downregulated (Fig. 6D). To substantiate these findings at the protein level, we examined the expression of key candidates RSPH4A, DNAAF1, and CFAP161 in RKO mouse retinas. Congruent with the transcriptomic and qPCR data, the protein levels of all three were consistently downregulated (Figs. 6E, 6F). Collectively, these results demonstrate that *Wdr34* deficiency leads to the coordinated downregulation of a suite of genes critically involved in axonemal integrity and microtubule-based transport. We propose that this collective impairment compromises the stability of the ciliary structure, thereby disrupting the efficient supply of proteins to the photoreceptor outer segment and ultimately triggering retinal degeneration.

### Subretinal Delivery of WDR34-Overexpressing AAV Partially Rescues Visual Function in RKO Mice

**Figure 7.**
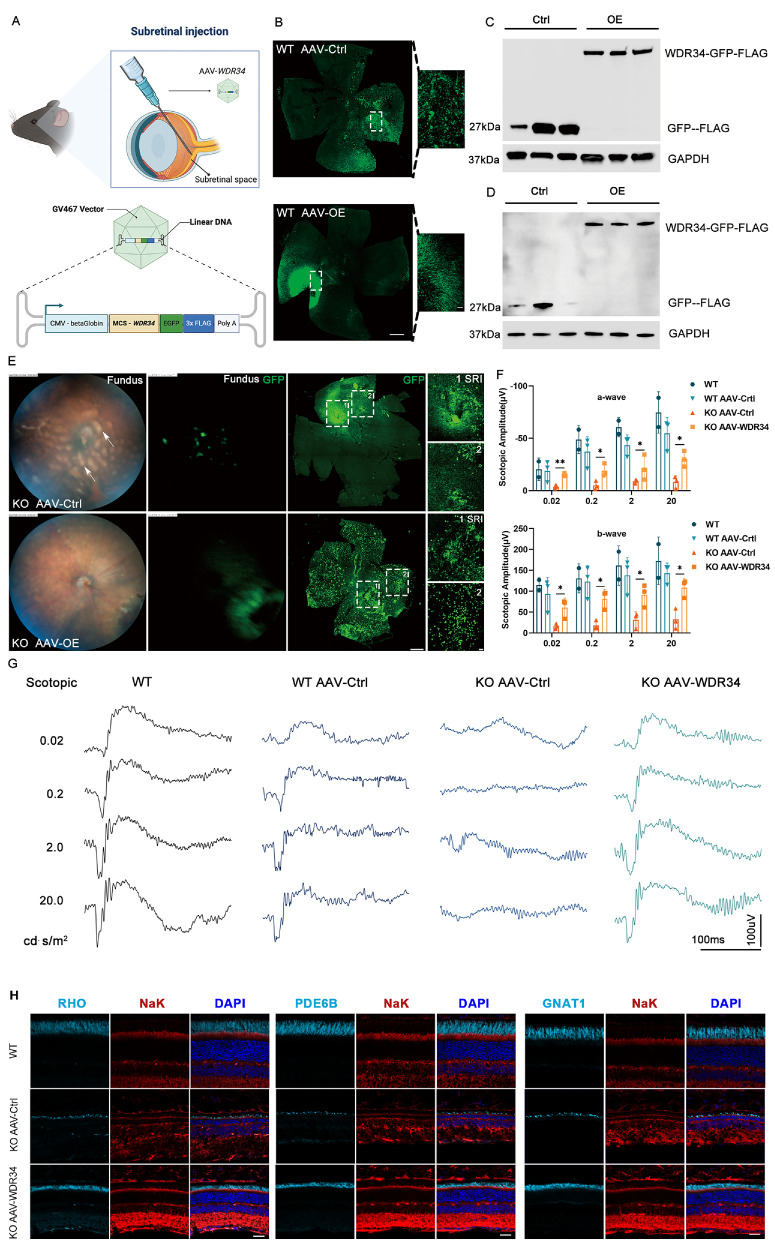
Subretinal Injection of AAV-WDR34 rescued retinal degeneration in *Wdr34^RKO^* mice. **(A)** Schematic illustration of subretinal injection (SRI) and construction of AAV vector for overexpressing WDR34. **(B)** Retinal flat-mount from a seven-week-old wild-type mouse one week after subretinal injection. *Green* represents EGFP, a fluorescent protein tag expressed after viral infection of photoreceptor cells. *Scale bar*: 500 µm. **(C)** Western blot analysis of HEK293T cells infected with AAV. **(D)** Western blot of retinal proteins collected 1 week after subretinal injection in WT mice. **(E)** Fundus examination and retinal flat-mount of *Wdr34^RKO^* mice at the age of five months old after SRI of AAV-Ctrl/OE. *Green* represents EGFP, a fluorescent protein tag expressed after viral infection of photoreceptor cells. Label 1 indicates the site of SRI; Label 2 indicates other parts of the retina, showing the spread effect. *Scale bars*: 500 µm for the fundus image; 50 µm for the retinal flat-mount. *White arrows* indicate regions of retinal degeneration. **(F)** Statistical analysis was performed for the amplitudes of the a-wave and b-wave under scotopic conditions (*n* = 3, two-way ANOVA followed by Tukey's post hoc test). *P < 0.05; **P < 0.01; ***P < 0.001; ns = no significant difference. Data are presented as mean ± SD. **(G)** ERG analysis under light-adapted conditions in 1.5-month-old WT and *Wdr34^RKO^* mice after subretinal injection of control and overexpression viruses AAV-Ctrl/WDR34, followed by rearing until four months of age. **(H)** Cryosections and immunofluorescence staining were analyzed in five-month-old WT mice, control (KO AAV-Ctrl) and treatment groups (KO AAV-WDR34). Outer segments were labeled in *light blue* (RHO, phosphodiesterase-6-β [PDE6B], and GNAT1), inner segments in *red* (Nak), and nuclei in *dark blue* (DAPI). *Scale bar*: 25 µm.

Significant progress has been made in the treatment of inherited retinal diseases through gene therapy.^19^ For instance, Leber congenital amaurosis (caused by RPE65 mutations has been successfully addressed.^20^^–^^22^ To offer patients greater hope and possibilities, we have developed a targeted therapeutic strategy for retinal degeneration caused by WDR34 deficiency. This strategy involves subretinal injection of adeno-associated virus (AAV-WDR34) carrying the WDR34 gene to achieve photoreceptor-specific gene compensation (Fig. 7A). Subretinal injection of AAV-WDR34 in wild-type mice resulted in robust GFP and FLAG expression (Figs. 7B, 7D), and transduction of HEK293T cells confirmed WDR34 overexpression (Fig. 7C).

In 1.5-month-old RKO mice, AAV-WDR34 treatment attenuated fundus pigmentary deposits (Fig. 7E) and significantly improved scotopic ERG responses at 14 weeks post-injection compared to AAV-Ctrl-injected controls (Figs. 7F, 7G). Meanwhile, wild-type mice injected with AAV-Ctrl showed no significant difference in a-wave and b-wave amplitudes compared to untreated WT mice, confirming the safety of the AAV vector in vivo (Figs. 7F, 7G). Further analysis using optical coherence tomography and immunofluorescence staining demonstrated a significant increase in the thickness of the ONL and the length of the OS (Supplementary Figs. S3A, S7H). Additionally, reduced GFAP immunostaining (Supplementary Fig. S3B) and corresponding Western blot results (Supplementary Figs. S3E, S3F) indicated downregulation of GFAP expression. Moreover, Western blot analysis revealed effective restoration of phototransduction-related proteins in the treatment group (Supplementary Figs. S3C, S3D). These results demonstrate that WDR34 gene supplementation partially rescues retinal structure and function in RKO mice.

## Discussion

In this study, phenotypic experiments on RKO and HKO demonstrated that the loss of *Wdr34* induces retinal degeneration phenotypes, such as impaired visual function and photoreceptor death. Further our transcriptomic sequencing revealed that *Wdr34* deficiency may disrupt intraflagellar transport in photoreceptors, compromising ciliary structural integrity, ultimately leading to photoreceptor degeneration. Importantly, subretinal delivery of AAV-mediated WDR34 overexpression partially restored visual function, highlighting the therapeutic potential of AAV-based gene therapy and offering a promising strategy for patients.

WDR34, a core component of the cytoplasmic dynein-2 complex, facilitates cargo transport within cilia.^23^ Western blot analysis of RKO mouse retinas revealed significant downregulation of disc-specific proteins (Figs. 3D, 3E) accompanied by reduced expression of cilia structural integrity and microtubule transport protein-associated genes (Fig. 6). These findings suggest potential impairment in the anterograde trafficking of key phototransduction components from IS to OS, which may underlie the observed visual dysfunction and retinal degeneration. However, because dynein-2 primarily mediates retrograde transport (OS to IS)^15^ along the cilium, the exact mechanism by which *Wdr34* deficiency disrupts disc membrane protein translocation remains unclear and warrants further investigation. WDR60 and WDR34 function as non-paired intermediate chains of the dynein-2 complex.^23^ Loss of Wdr60 leads to neurodevelopmental abnormalities, which can be rescued by overexpression of a stabilized α-tubulin K40Q mutant (an acetylation-mimicking variant).^24^ Importantly, RKO mice displayed reduced photoreceptor cilium number and shortened cilium length (Fig. 4), findings that were further corroborated by cellular experiments (Figs. 5G–I, Supplementary Fig. S3E–I). These results suggest that WDR34 deficiency may compromise the structural integrity of photoreceptor ciliary axonemes, resulting in reduced tubulin acetylation. This pathological alteration could lead to OS shortening and subsequent retinal degeneration. This may represent one potential mechanism underlying impaired disc membrane protein trafficking.

Vision initiates in the OS of photoreceptors, where light absorption triggers phototransduction cascades that relay signals to the nervous system. Disc membrane proteins are indispensable for this phototransduction process.^25^^,^^26^ Our experimental data demonstrate significant downregulation of four critical disc membrane proteins (RHO, GRK1, PRPH2, and GNAT1) in RKO mice (Figs. 3D, 3E). Dysregulation of these proteins is well-established to cause severe retinal degeneration through distinct pathogenic mechanisms. RHO, the first gene identified in autosomal dominant RP, undergoes mutation-induced misfolding and mislocalization, leading to aberrant signal transduction and eventual retinal degeneration.^27^^,^^28^ GNAT1 mutations are associated with congenital stationary night blindness and impair rod photoreceptors' ability to activate phosphodiesterase-6, thereby disrupting cGMP hydrolysis and precipitating retinal degeneration.^29^^–^^31^ GRK1 mutations cause Oguchi disease (a night blindness disorder) by preventing proper deactivation of photoactivated RHO, resulting in profoundly delayed rod cell recovery and subsequent retinal degeneration.^32^^,^^33^ PRPH2, essential for OS disc morphogenesis and structural maintenance, causes severe OS disorganization and retinal degeneration when mutated.^34^^,^^35^

Gene therapy using AAV vectors has emerged as an effective strategy for treating retinal degeneration caused by genetic mutations.^5^^,^^36^^–^^38^ In 2008, the first successful clinical application of RPE65 gene augmentation therapy in patients with Leber congenital amaurosis or early-onset RP demonstrated both the safety and efficacy of this approach, leading to the first FDA-approved gene therapy for retinal disease.^20^^–^^22^ In this study, we show that subretinal delivery of AAV-WDR34 effectively restores visual function and retinal structure in RKO mice (Figs. 7G, 7H), offering therapeutic potential for patients. However, despite being one of the most promising treatments for inherited retinal diseases, gene therapy still faces several limitations, including the requirement for early-stage intervention prior to significant cellular degeneration, the limited cargo capacity of AAV vectors, dominant-negative mechanisms, and high economic costs.^5^ Therefore continued development of diversified therapeutic approaches remains crucial, including small molecule-based neuroprotective agents, retinal microenvironment-targeted anti-inflammatory therapies, retinal progenitor cell transplantation, retinal prosthetic implantation, and optogenetic visual pathway remodeling.^39^^–^^41^

Although our study confirms that WDR34 deficiency leads to ciliary disintegration and photoreceptor degeneration, the direct molecular pathway linking ciliary defects to the transcriptional downregulation of various photoreceptor genes remains unresolved. A key question persists: does the loss of WDR34 disrupt the transport of transcriptional regulators, directly leading to reduced mRNA levels of various photoreceptor genes, or is the transcriptional downregulation a secondary consequence of the progressive photoreceptor degeneration? Previous studies have established that ciliary damage primarily disrupts the trafficking of critical proteins from the IS to the OS, and that the ensuing failure to supply OS proteins directly triggers degeneration.^42^^–^^44^ Therefore we favor a model wherein WDR34 deficiency first impedes the transport of essential OS proteins, initiating progressive OS shortening. The resulting structurally diminished OS possesses a reduced capacity for housing phototransduction proteins. Consequently, the downregulation of these phototransduction genes is likely a secondary, homeostatic adaptation to the collapsed OS structure, rather than the primary cause of degeneration. Future studies aimed at distinguishing the primary transport defects from secondary transcriptional responses will be crucial to fully resolve this temporal sequence of events.

The present study demonstrates that WDR34, as a dynein-2 intermediate chain 2, plays a critical role in maintaining photoreceptor function through its mediation of intraflagellar transport and ciliary integrity, which are essential for cellular architecture, disc membrane protein trafficking, and the phototransduction system. These findings significantly advance our understanding of WDR34's function and mechanistic roles in retinal biology. Importantly, AAV-WDR34-mediated gene therapy effectively delayed disease progression in WDR34-associated retinal degeneration, offering a promising therapeutic strategy for affected patients.

## Supplementary Material

Supplement 1
