# Supplementary material for: Rod-Cone Dystrophy Related WDR34 Is Essential for Ciliary Integrity and Survival of Mammalian Photoreceptor Cells
Source: Invest Ophthalmol Vis Sci. 2026 Jan 12;67(1):26. doi: 10.1167/iovs.67.1.26 (PMC12810420; doi:10.1167/iovs.67.1.26)
Supplement: Supplement 1 [file iovs-67-1-26_s001.pdf]

Rod-cone dystrophy related WDR34 is essential for ciliary integrity and survival of mammalian photoreceptor cells

Rong Zou[1,2#], Jinrui Cai[1#], Lin Fan[1#], Luning Liu[3#], Can Chen[1], Guangyi Chen[1], Xian Yang[3*], Kuanxiang Sun[1,2*], Xianjun Zhu[1,2*]

[1]The Sichuan Provincial Key Laboratory for Genetic Diseases, Center for Medical Genetics, Sichuan Provincial People's Hospital, School of Medicine, University of Electronic Science and Technology of China, Chengdu, Sichuan, 610072, China;

[2]Sichuan-Chongqing Joint Key Laboratory for Pathology and Laboratory Medicine, Jinfeng Laboratory, Chongqing, 401329, China;

[3]Department of Ophthalmology, The Affiliated Hospital of Qingdao University, Qingdao, Shandong, 266000, China.

Supplementary data include supplemental figure S1-S3 and table S1-S3.

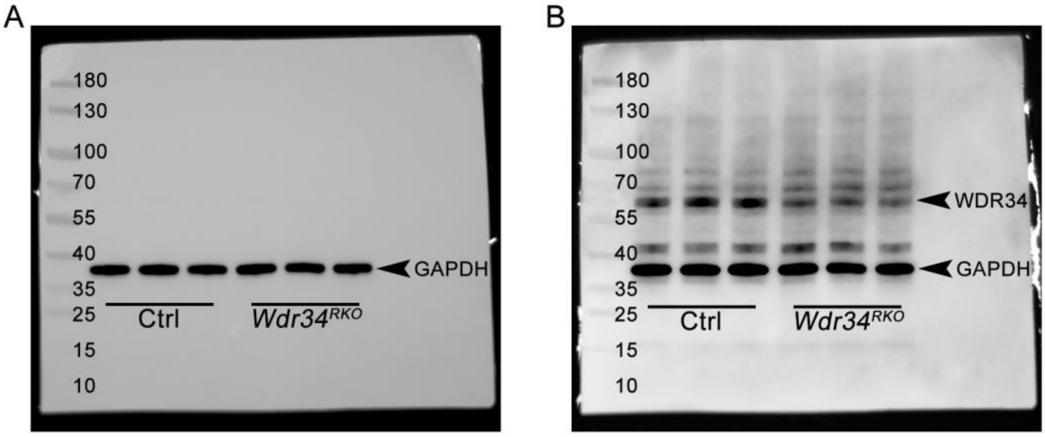

Figure S1. Verification of WDR34 knockout efficiency in RKO mice.

A) Protein samples were obtained from the retinas of 2-month-old Ctrl and RKO mice. The full blot image of the GAPDH antibody was displayed. **B)** The full blot image shows the WDR34 antibody and the GAPDH antibody band.

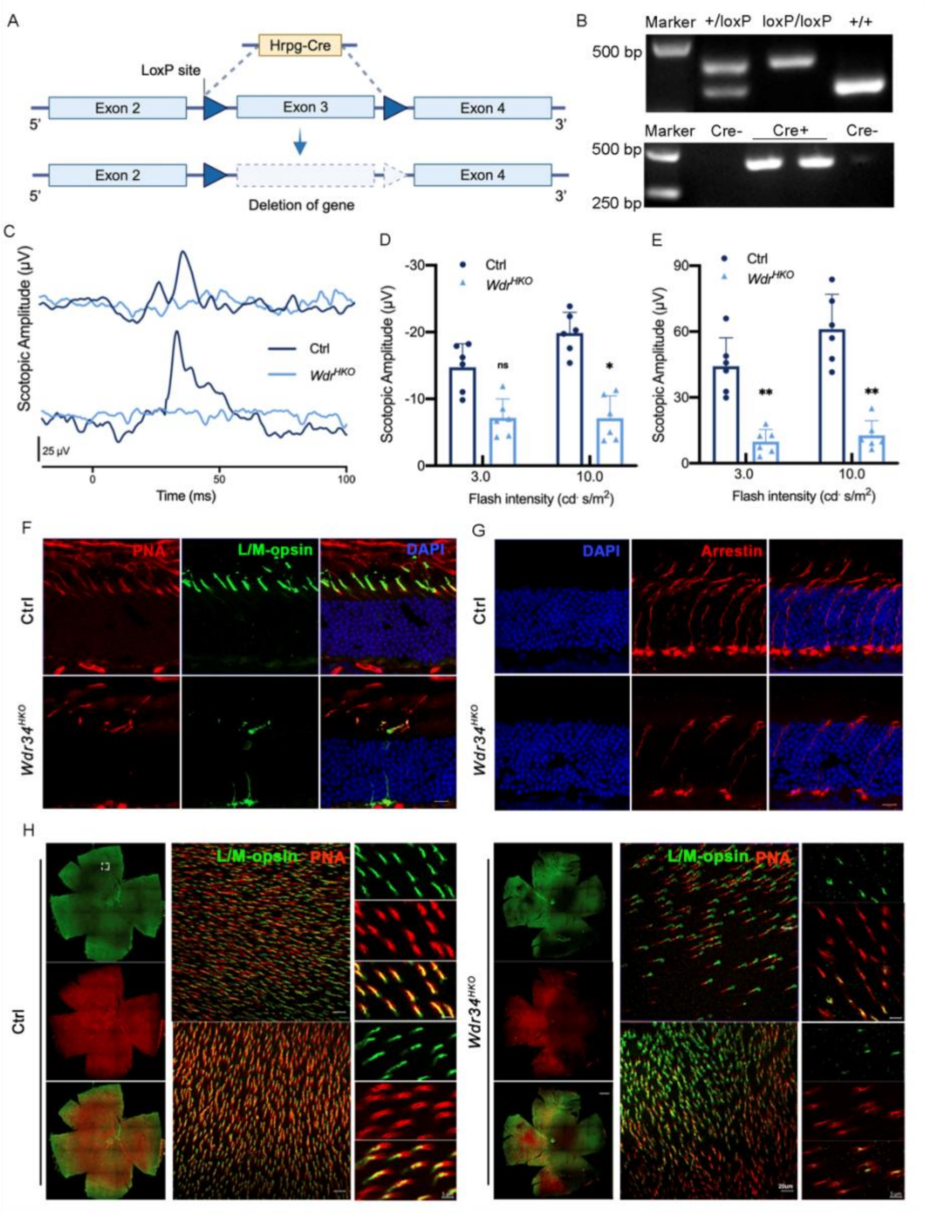

Figure S2. Knockout of *Wdr34* resulted in cone cell death and impaired M-opsin transport in *Wdr34*^{HKO} mice.

A) Schematic diagram of conditional knockout construction of cone specific knockout model *Wdr34*^{HKO}. **B)** Genotyping results of *Wdr34*^{HKO} mice. **C)** Representative electroretinogram (ERG) traces corresponding to responses elicited by light-adapted conditions at flash intensities of 3.0, 10.0 cd·s/m^2 at 4-month-old in *Wdr34*^{HKO} mice. **D, E)** Statistical analysis was performed for amplitudes of a-wave (**D**) and b-wave (**E**) under light-adapted conditions in mice; sample size n = 6, Tukey's multiple comparisons ANOVA. *$p < 0.05$, **$p < 0.01$, ns = no significance. **F)** Representative immunofluorescence images of Opsin (green) and PNA (red) in retinas from 8-month-old Ctrl and *Wdr34*^{HKO} mice was shown. Nuclei were counterstained with DAPI (blue). Scale bar: 20 μm. **G)** Representative immunofluorescence labeling of retinal cryosections from

Ctrl and $Wdr34^{HKO}$ mice at the age of 8 months using a cone Arrestin (red) and DAPI (blue). Scale bar: 20 μm. **H)** Immunofluorescence labeling of retinal flat-mounts from 8-month-old $Wdr34^{HKO}$ mice using peanut agglutinin (PNA) (red) to label Cre-positive cells, M-Opsin antibody (green) to label cone outer segments, and DAPI (blue) for nuclear counterstaining. Representative images are presented in the middle panel, with corresponding cropped and magnified views shown in the right panel. Scale bars from left to right: 500 μm, 50 μm, 5 μm.

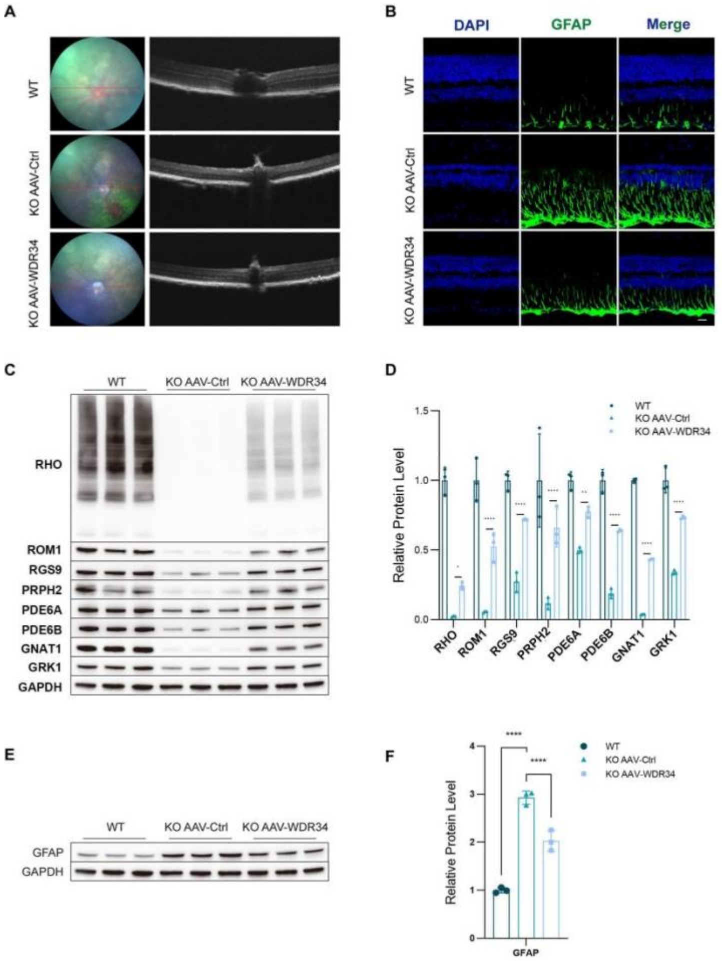

Figure S3. Therapeutic intervention alleviated the progression of retinal degeneration in $Wdr34^{RKO}$ mice.

A) Optical coherence tomography (OCT) was analyzed in 5-month-old WT mice, control (KO AAV-Ctrl) and treatment groups (KO AAV-WDR34). Scale bar: 100 μm. **B)** Cryosections and immunofluorescence staining were analyzed in 5-month-old WT mice, control (KO AAV-Ctrl) and treatment groups (KO AAV-WDR34). Astrocytes were labeled in green (GFAP) and nuclei in blue (DAPI). Scale bar: 25 μm. **C, D)** Western Blot (**C**) and quantitative comparison (**D**) of protein expressions of RHO, ROM1, RGS9, PRPH2, PDE6A, PDE6B, GNAT1 and GRK1 in retinas from 5-month-old control (KO AAV-Ctrl) and treatment groups (KO AAV-WDR34). GAPDH served as

the loading control (n=3). **E, F)** Western Blot (**E**) and quantitative comparison (**F**) of protein expressions of GFAP in retinas from 5-month-old control (KO AAV-Ctrl) and treatment groups (KO AAV-WDR34). GAPDH served as the loading control (n=3). All comparisons were performed using Dunnett's multiple comparison test. *p < 0.05; **p < 0.01; ***p < 0.001; ****p < 0.0001; ns = no significant difference. Data are presented as the mean ± SD.

Table S1. Polymerase chain reaction-based primers for mouse genotyping.

Primer name	Primer sequence
Rod-Cre-F	GAACGCACTGATTTCGACCA
Rod-Cre-R	GCTAACCAGCGTTTTCGTTC
HRGP-Cre-F	GAACGCACTGATTTCGACCA
HRGP-Cre-R	GCTAACCAGCGTTTTCGTTC
Wdr34^{flox}-F	GGCCTGCCGATTCATGATA
Wdr34^{flox}-R	TTGTCCTGCTCAACACCTAG

Table S2. Real-time quantitative PCR primers.

Species	Primer name	Primer sequence
Mouse	*Wdr34*-qPCR-F	TGTCTGCACACCCTGGTCTA
Mouse	*Wdr34*-qPCR-R	TCCATCATCTAGCCGACCAT
Mouse	*Rho*-qPCR-F	CTTCCCCATCAACTTCCTCA
Mouse	*Rho*-qPCR-R	GAATCCTCCGAAGACCATGA
Mouse	*Prph2*-qPCR-F	ACGGACTCAAGAATGGGATG
Mouse	*Prph2*-qPCR-R	TAGCGATTGCTGATCCACTG
Mouse	*Pde6b*-qPCR-F	TGCAGCACTTTTTGAACTGG
Mouse	*Pde6b*-qPCR-R	AATCCTCCAGAAGGCTGTCA
Mouse	*Grk1*-qPCR-F	CGGGGCAGTTTTGACGGAA
Mouse	*Grk1*-qPCR-R	AGCTGAGGTTGTCACGGAGA
Mouse	*Gnat1*-qPCR-F	TGCAATCATCGCTACTTCGC
Mouse	*Gnat1*-qPCR-R	GTCGCATGTTAAGCTCCAGG
Mouse	*Rsph4a*-qPCR-F	CCTGTCTACGGGAGACCTCAC
Mouse	*Rsph4a*-qPCR-R	GTCAGCGATTCCATTAGCGGT

Mouse	*Dnaaf11*-qPCR-F	AAAGCACCATGGGCCGAATC
Mouse	*Dnaaf11*-qPCR-R	TAAGTCCCGGCACCATTTGT
Species	Primer name	Primer sequence
Mouse	*Cfap221*-qPCR-F	AACTATCGTCTGCAGGGAAAT
Mouse	*Cfap221*-qPCR-R	AGCTTGGCATAAACCCTGGAT
Mouse	*Dynlt4*-qPCR-F	GCACCCTCATACCGCTTAGAAC
Mouse	*Dynlt4*-qPCR-R	CATAGTGCCTGCACCAGTTTCC
Mouse	*Kif2c*-qPCR-F	ATGGAGTCGCTTCACGCAC
Mouse	*Kif2c*-qPCR-R	CCACCGAAACACAGGATTTCTC
Mouse	*Kif19b*-qPCR-F	GATGTCATCAGAGCGAGCCT
Mouse	*Kif19b*-qPCR-R	TCCTTGGGTTCCATCTCCCT
Mouse	*Kif18b*-qPCR-F	TATGCTGACCGTGCCAAGGAGA
Mouse	*Kif18b*-qPCR-R	GAGTTTCTCCCTCAGGAAGGCT
Mouse	*Cfap161*-qPCR-F	TGAGAGGAGACCTGAGCCTG
Mouse	*Cfap161*-qPCR-R	CAACCACTTGGCCCATTTCG
Mouse	*Gapdh*-qPCR-F	TGTGTCCGTCGTGGATCTGA
Mouse	*Gapdh*-qPCR-R	TTGCTGTTGAAGTCGCAGGAG
Human	WDR34-qPCR-F	ATCCGCTGGGAGACGAAAAG
Human	WDR34-qPCR-R	GGGCGTCCACATGATTCCT
Human	GAPDH-qPCR-F	CCATGGGTGGAATCATATTGGA
Human	GAPDH-qPCR-R	TCAACGGATTTGGTCGTATTGG

Table S3. Antibodies used in the current study.

Name of Antibodies	Dilution (IHC/ICC)	Dilution (WB)	Vendor name
Rabbit Anti-RHO	1/200		Proteintech, USA
Rabbit Anti-RHO		1/2000	CST, USA
Rabbit Anti-PRPH2		1/2000	Proteintech, USA
Rabbit Anti-PED6B	1/200	1/2000	Proteintech, USA
Mouse Anti-NaK ATPase	1/200		ThermoFisher, USA
Rabbit Anti-GNAT1		1/2000	Abcam,UK
Rabbit Anti-GRK1		1/2000	Abcam,UK
Rabbit Anti-WDR34	1/1000	1/500	Novusbio, USA
Rabbit Anti-RSPH4A		1/2000	Proteintech, USA
Rabbit Anti-DNAFF11		1/2000	Proteintech, USA
Rabbit Anti-CFAP161		1/2000	Proteintech, USA
Mouse Anti-Acetylated α- tubulin	1/500	1/2000	Sigma Aldrich, USA
Rabbit Anti-CEP164	1/200		CST, USA
Mouse Anti-γ-tubulin	1/2000		Abcam,UK
594-conjugated F-actin	1/2000		Yeasen, China
Rabbit Anti-m-OPSIN	1/500		Millipore, USA
594-conjugated PNA	1/200		Vector laboratories, USA
Rabbit Anti-Cone Arrestin	1/500		Sigma Aldrich, USA
Rabbit Anti-GAPDH		1/5000	Proteintech, USA
Anti-rabbit HRP-conjugated secondary antibody		1/5000	Proteintech, USA
Anti-mouse HRP-conjugated secondary antibody		1/5000	Proteintech, USA
Goat-Anti-rabbit 647	1/500		Invitrogen, USA
Goat-Anti-rabbit 488	1/500		Invitrogen, USA
Goat-Anti-mouse 594	1/500		Invitrogen, USA
DAPI	1/1000		Sigma Aldrich, USA
